# Stable Sulforaphane Targets the Early Stages of Osteoclast Formation to Engender a Lasting Functional Blockade of Osteoclastogenesis

**DOI:** 10.3390/cells13020165

**Published:** 2024-01-16

**Authors:** Polymnia Louka, Isabel R. Orriss, Andrew A. Pitsillides

**Affiliations:** Skeletal Biology Group, Comparative Biomedical Sciences, Royal Veterinary College, London NW1 0TU, UK; plouka@rvc.ac.uk (P.L.); iorriss@rvc.ac.uk (I.R.O.)

**Keywords:** sulforaphane, osteoclasts, osteoclastogenesis, NRF2 and NF-KB

## Abstract

Sulforaphane, the native but unstable form of SFX-01, is an antioxidant that activates the NRF2 and inhibits the NF-KB pathways to achieve its actions. Resolving the mechanism(s) by which SFX-01 serves to control the various osteoclastogenic stages may expose pathways that could be explored for therapeutic use. Here we seek to identify the stage of osteoclastogenesis targeted by SFX-01 and explore whether, like SFN, it exerts its actions via the NRF2 and NF-KB pathways. Osteoclasts generated from the bone marrow (BM) of mice were cultured with SFX-01 at different timepoints to examine each phase of osteoclastogenesis separately. This showed that SFX-01 exerted actions throughout the process of osteoclastogenesis, but had its largest effects in the early osteoclast precursor differentiation stage. Thus, treatment with SFX-01 for the duration of culture, for the initial 3 days differentiation or for as little as the first 24 h was sufficient for effective inhibition. This aligned with data suggesting that SFX-01 reduced DC-STAMP levels, osteoclast nuclear number and modified cytoskeletal architecture. Pharmacological regulation of the NRF2 pathways, via selective inhibitors/activators, supported the anti-osteoclastogenic roles of an SFX-01-mediated by NRF2 activation, as well as the need for tight NF-KB pathway regulation in osteoclast formation/function.

## 1. Introduction

Several clinical conditions including osteoporosis, Paget’s disease and other cancer-associated rarer bone diseases are characterised by bone loss and increased osteoclast activity [1,2]. The targeting of osteoclasts in skeletal pathobiology has thus been explored and has generated pharmacological agents capable of modulating osteoclastogenesis, resorption and ensuing bone loss. These agents, including bisphosphonates, selective oestrogen receptor modulators and anti-RANKL antibodies, are effective in restricting osteoclastic bone resorption but nonetheless exert undesirable side effects such as impaired bone remodelling [2]. For this reason, there remains demand for alternative approaches to control osteoclast formation and bone resorptive activity; antioxidant agents have been attributed with such anti-resorptive therapeutic potential [3,4].

Sulforaphane (4-methylsulfinylbutyl isothiocyanate, SFN) is a phytochemical produced from a biologically inactive precursor, glucoraphanin, during the biting and chewing of certain plant sources [5,6,7,8,9]; this precursor is found in cruciferous vegetables and many nutraceuticals [10]. SFN is usually well tolerated and is known to exert potent antioxidant effects by modifying cytoplasmic KEAP1 conformation, releasing NRF2 for nuclear translocation and the transcriptional activation of genes with antioxidant response elements (ARE) in their promoters [11,12,13,14,15]. While cellular SFN uptake leads to an initial burst in ROS production, its rapid activation of the KEAP1-NRF2-ARE system induces an overall antioxidative or anti-inflammatory profile of cellular behaviours [16,17,18]. SFN is, however, highly unstable, rapidly degraded by heat and light and highly sensitive to oxygen and pH [19]. SFN pharmacokinetics are therefore highly variable and inconsistent, making it difficult to ensure effective levels of an attractive therapeutic for a sufficient duration, and its clinical advances have been restricted.

Technology for synthesising and concurrently stabilising SFN has, therefore, been developed to unlock its pharmaceutical potential. This has yielded a stable alpha-cyclodextrin–SFN complex, Sulforadex^TM^ (SFX-01), that is safe, non-toxic and well tolerated in humans, but is rarely used compared to SFN. SFX-01 is, nonetheless, known to recapitulate the vital targeting of NRF2 and STAT3 [20] by endocrine resistant stem-like cells to inhibit lung metastasis in estrogenic receptor (ER)-positive breast cancer [20]. This clinical candidature of SFX-01 is reinforced by its efficacy in a mouse model of relapsing experimental autoimmune encephalomyelitis in which it reduced residual disability, decreased the maximum severity of relapses and improved recovery [21]. Beneficial effects of SFX-01 have also been observed in a preclinical human glioblastoma model [22]. This has led to the examination of SFX-01 as a therapeutic in Phase 2 trials in ER-positive metastatic breast cancer and spontaneous subarachnoid haemorrhage patients [23].

Few studies have, however, explored the effects of SFX-01 in skeletal pathophysiology. SFX-01 administration has been found to reduce the levels of gait asymmetry that emerge spontaneously in a natural osteoarthritis model in STR/Ort mice [24], in which such asymmetries are otherwise predictive of disease progression [25]. This was accompanied by increases in trabecular bone mass and indices of bone strength, and higher procollagen type-I N-propeptide (PINP, bone formation) and, notably, lower type-I collagen cross-linked C-telopeptide (CTX-1, bone resorption) serum markers; changes that were not explained by possible direct in vitro effects of SFX-01 on primary osteoblasts, in which only modest, if any, effects were detected on mineralised nodule formation at 10–100 nM SFX-01 [24]. It has also been shown that the divergent basal bone mass found in different mouse strains is linked to in vitro osteoclastogenic potential and that this aligns with sensitivity to SFX-01 [26]. These data suggest that SFX-01 represents an alternative approach to controlling osteoclast formation and resorptive activity.

There are extensive studies using SFN that support this notion. SFN inhibits RANKL-induced NF-KB activation in RAW 264.7 cells [27] and restricts the expression of the cell fusion molecules, DC-STAMP and OC-STAMP, by inducing STAT1 phosphorylation [27]. This aligns with microarrays of RAW 264.7 cells and primary osteoclasts in which SFN lowered OSCAR, NFATc1 and TRAP, DC-STAMP and OC-STAMP mRNA levels whilst STAT1, a macrophage and osteoclast fusion regulator, was instead increased [28]. Hyeon et al., (2013) explored the links between SFN and NRF2, finding both more osteoclast multinucleation and resorption, with raised oxidative stress, in cells from NRF2 knockout mice, and an SFN-related inhibition of osteoclast differentiation in cells from wildtype mice [29], suggesting that SFN likely acts through NRF2 to suppress osteoclastogenesis. In vivo data showing elevated metaphyseal cancellous bone volume in both normal and ovariectomised mice treated with SFN indicate that SFN can indeed reduce bone loss due to oestrogen deficiency [30].

These data indicate that SFN and SFX-01 may have therapeutic value in skeletal diseases where bone resorption or accelerated remodelling is causal. It is therefore necessary to define whether SFX-01 also exerts antiresorptive actions and, if so, which osteoclastogenic stage(s) it targets and its mechanism(s) of action. This will verify its therapeutic potential and likely identify alternative antiresorptive candidates.

It is appropriate to also reflect on the methods used to study osteoclastogenesis. Initial advances involved cell isolation from fragmented neonatal bones and their deposition onto bone/dentine to allow for pit excavation [31]. Long-term in vitro osteoclast formation now uses hematopoietic precursors from marrow, the spleen or peripheral blood [32,33]; supplementation with, for example, PTH, M-CSF and RANKL with similar deposition onto bone/dentine. This has the advantage of allowing the separate examination of key osteoclast formation, activation and resorption phases, which is impossible on plastic. Almost all studies reporting the inhibitory effects of SFN on osteoclastogenesis have relied, however, on RAW 264.7 cell lines or primary cells seeded onto plastic, showing the direct effects on differentiation but not resorption [27,28,34]. One study elegantly extended this to study cells seeded on dentine, reporting that SFN reduces the resorption pit number but not the area of dentine resorbed per osteoclast, concluding that SFN strongly inhibited osteoclast formation at an early precursor differentiation stage [27]. Here, using primary osteoclasts on dentine, we examine how SFX-01 influences each of the key osteoclast formation, activation and resorption phases, and explore whether, like SFN, it exerts its actions via the NRF2 and NF-KB pathways.

## 2. Materials and Methods

### 2.1. Animals

All the procedures conducted in the facility were in accordance with the Animals Act (Scientific Procedures) 1986 and approved by the Royal Veterinary College (RVC) Research Ethics Committee. Bone marrow (BM) cells were isolated from C57BL/6, CBA, and STR/Ort strains. Mice were housed at 21 ± 2 °C with 12-h light/dark cycles and free access to food and water.

### 2.2. Osteoclast Cell Culture

Osteoclast precursors were isolated from the bone marrow of mouse long bones (6–8 weeks old) and cultured using a 3-step protocol, as described previously [26,33]. Briefly, the resultant BM suspension was then centrifuged at 1500 rpm and first resuspended in MEM which was initially supplemented with 10% foetal calf serum (FCS), 2 mM l-glutamine, 100 U/mL penicillin, 100 μg/mL streptomycin and 0.25 μg/mL amphotericin, 100 nM prostaglandin E_2_ (PGE_2_) and 50 ng/mL M-CSF. The cell suspension was cultured for 24 h in 75 cm^2^ flasks in 5% CO_2_/95% atmospheric air. The next day (Day 1), the non-adherent cell suspension was removed, centrifuged and resuspended in MEM containing 10% FCS, 2 mM l-glutamine, 100 U/mL penicillin, 100 μg/mL streptomycin and 0.25 μg/mL amphotericin,100 nM PGE_2_ and 200 ng/mL M-CSF, as well as 5 ng/mL RANKL (R&D Systems Europe Ltd., Abingdon, UK)—referred to as S2MEM, see below—in a step designed to promote osteoclast commitment. Cells were plated onto 5 mm diameter dentine discs (10^6^ cells/disc) in 96-well plates and incubated overnight at 37 °C/5% CO_2_ to allow for the attachment of osteoclast precursors. After 24 h (Day 2), the dentine discs were transferred to six-well plates (3–4 discs/well in 3 mL S2MEM) and maintained for 3 days. Finally, S2MEM was acidified to pH 6.9 (Day 5) by addition of HCl to activate osteoclast resorption for 48 h (Day 7).

**Figure 1:**
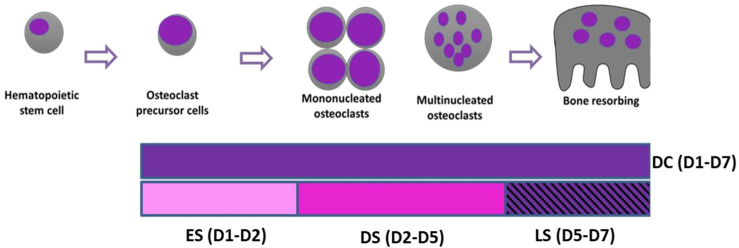
Schematic illustration of dosing regimen to study the effects of SFX-01 on defined stages of osteoclast differentiation, maturation and resorption. **DC**: treatment throughout the duration of the culture (D1–D7). **ES**: SFX-01 was added on D1 after plating and removed after 24 h on D2 (early-stage treatment, D1–D2). **DS**: SFX-01 was added on D2 and removed on D5 (differentiation stage treatment, D2–D5). **LS**: SFX-01 was added only during later resorption stage on D5 until the termination of the culture on D7 (later stage treatment D5–D7).

To establish stages at which SFX-01 exerts actions on osteoclast formation, differentiation and resorption, cells from each marrow isolation were cultured in osteoclastogenic medium, with or without 2.5 μΜ SFX-01, during very specifically defined and different time points; 2.5 μΜ was used as it has been previously showed to be the optimal concentration to inhibit osteoclastogenesis in osteoclasts derived from STR/Ort mice [26]. The protocol for this evaluation of ‘staged’ mouse osteoclast formation, differentiation and resorption aligns with the very defined step-wise modification in culture conditions (media and culture substrates) documented previously (see [33]). Cells were exposed to SFX-01 (or vehicle) either: (i) for the entire duration of post-plating culture from day 1–7 (during culture; hence DC; D1–7); (ii) for only the early stage (ES) where SFX-01 was added on D1 post-plating and removed 24 h thereafter on D2 (hence ES; D1–D2); (iii) during only the differentiation stage (DS), excluding the resorption phase, where SFX-01 was added on D2 and was retained in all culture medium changes until it was removed on D5 (hence DS; D2–D5); or (iv) during only the later ‘resorption’ stage (LS) where SFX-01 was added on D5 until the termination of the culture on D7 (hence LS; D5–D7) (Figure 1). All cultures were replenished with fresh control medium during the periods not supplemented with SFX-01, and in all cases, experimental protocols were terminated on day 7 of culture.

For some experiments, pharmacological inhibitors/activators of NRF2 and NF-KB were initially used alone for the duration of the culture (DC), at one of two different concentrations (1 μM and 10 μM). For NRF2 activation and inhibition, RA839 and Trigonelline (Trig) were used, respectively; while for NF-KB activation and inhibition, Betulinic acid (BA) and SC-514 were used (all at 1 μM or 10 μM). Each compound was also added in conjugation with SFX-01 (2.5 μM) during the early stage (ES; from D1–D2) of culture, for 24 h after plating (at which time SFX-01 was found to be most effective).

All experiments used bone marrow cells derived from STR/Ort mice, with the sole exception of the experiment seeking to identify dose-dependent effects of pharmacological activators/inhibitors of the NRF2 and NF-KB pathways in which CBA and C57BL/6 mice were used instead.

### 2.3. Analysis of Osteoclast Formation and Bone Resorption

Discs containing adherent cells/osteoclasts were fixed in 2% glutaraldehyde and stained to demonstrate tartrate-resistant acid phosphatase (TRAP) activity. Osteoclasts were defined as TRAP-positive cells containing two or more nuclei and/or clear evidence of resorption pit formation [33]. Osteoclast number and the area resorbed on each disc were assessed ‘blind’ by transmitted light microscopy and reflective light microscopy and dot-counting morphometry using Image J, respectively [33].

### 2.4. Immunocytochemistry

Osteoclasts grown in tissue culture 96-well plates for 6 days were treated with SFX01 as described above at specific, defined timepoints (ES, DS or DC), and then fixed with 4% paraformaldehyde in PBS for 20 min. Cells were washed twice with 0.05% PBS Tween 20 (PBS-T) and then exposed to 1% BSA in PBS-T for 30 min to block non-specific binding sites, prior to incubation for 2 h with an anti-DCSTAMP antibody (clone 1A2 primary antibody, 1:500). Fluorescently tagged goat anti-mouse IgG was used as secondary antibody (1:1000) and added to for 1 h at room temperature in the dark. For F-actin ring staining, phalloidiniFluor488 reagent was added for 30 min at room temperature. After washing with PBS, DAPI was used to stain nuclei. Cells were imaged using a DMIRB microscope to allow for simultaneous imaging of three fluorescent labels.

### 2.5. ELISA for 4-HNE

The levels of lipid peroxidation, as a stable marker for oxidative stress, were measured using the 4-hydroxynonenal (4-HNE) ELISA kit (ab238538, Abcam., Cambridge, MA, USA) according to the manufacturer’s instructions. BM cells extracted from STR/Ort mice were cultured for 6 days on plastic discs as described above and SFX-01 was only added during the early (ES) and/or differentiation (DS) stages along with an appropriate untreated control group. The cultures were replenished with fresh control medium during the periods not supplemented with SFX-01. The absorbance was read immediately on the microplate reader at 450 nm. The concentrations of the 4-HNE adduct were quantified through a comparison with the absorbance of a known 4-HNE-BSA standard curve.

### 2.6. Statistics

All statistical analyses were performed using GraphPad Prism 8. To show the difference between two groups, a two-tailed paired *t*-test was performed. Between three or more groups, a repeated measures one-way analysis of variance or a repeated measures mixed-effects model was performed with Fisher’s LSD post hoc analysis. Each individual reported set of data represents *n* = 4–5 biological ‘experimental’ replicates using cells derived from different animals. Individual experiments comprised 6–8 technical replicates (number of discs) per group.

## 3. Results

### 3.1. SFX-01 Preferentially Targets the Early Stages of Osteoclastogenesis but Exerts Lasting Restriction upon Osteoclast Resorptive Function

**Figure 2:**
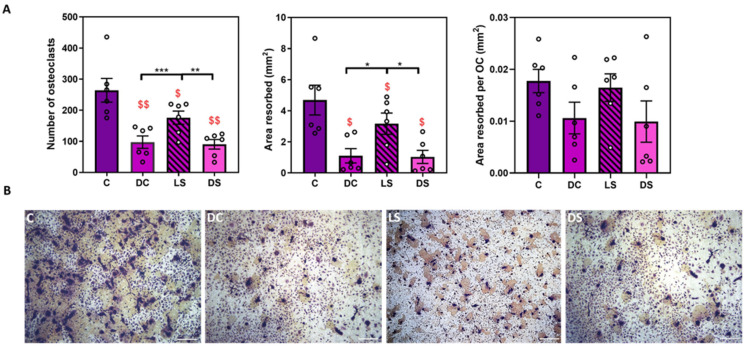
SFX-01 targets osteoclast formation. (**A**) We added 2.5 μΜ SFX-01at different time points during osteoclast culture, either throughout the culture (DC; D1–D7) or at later resorption stage (LS; D5–D7) or (differentiation stages (DS; D2–D5): (**A**) TRAP+ osteoclast numbers (**top left**), area resorbed (**middle**), area resorbed/OC (**top right**, all mean ± SE) from STR/Ort mice. (**B**) Representative reflective light images of osteoclasts at different stages after SFX-01 addition. $ and $$ denote *p* ≤ 0.05 and *p* ≤ 0.01 difference from control, respectively. *, ** and *** denote *p* ≤ 0.05, *p* ≤ 0.01 and *p* ≤ 0.001 differences within groups DC, LS, DS, C, respectively. Combined data from *n* = 6 separate experiments, with 6–8 replicates/condition/experiment.

Initial studies explored the effects of SFX-01 supplementation throughout the culture (DC; D1–D7) or only during the late (LS; D5–D7) or differentiation stages (DS; D2–D5; Figure 1). These data indicate that SFX-01 inhibited the osteoclast number and total area resorbed irrespective of treatment duration (Figure 2). These results also show that, whilst treatment at the late stage (D5–D7) was inhibitory, the size of the effects was less marked than when SFX-01 was present from days 1–7 (DC) or D2–D5 (DS). SFX-01 failed to exert significant modification in the area resorbed/osteoclasts under any of the dosing regimens (Figure 2).

### 3.2. SFX-01 Exposure Solely at a Really Early Stage (D1–2) Is Sufficient for Lasting Inhibition of Osteoclastogenesis/Resorption

**Figure 3:**
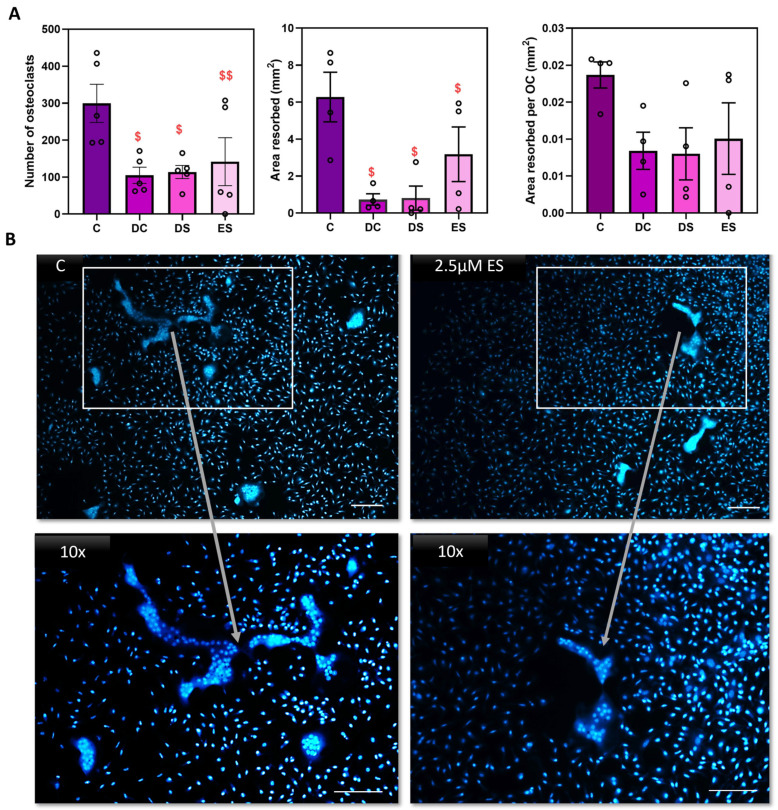
Exposure to SFX-01 for 24 h is sufficient to inhibit osteoclastogenesis. (**A**) We added 2.5μΜ SFX-01 at defined timepoints during osteoclast culture either throughout the culture (DC; D1–D7) or at differentiation (DS; D2–D5), or only at early stage (ES; D1–D2) for 24 h after plating on dentine and imaged and quantified conventionally: TRAP+ osteoclast numbers (**top left**), area resorbed (**middle**), area resorbed/OC (**top right**, all mean ± SE) from STR/Ort mice. $ and $$ denote *p* ≤ 0.05 and *p* ≤ 0.01 difference from control, respectively. Combined data from *n* = 6 experiments, with 6–8 replicates/condition. (**B**) We added 2.5 μΜ SFX-01 during the early stage (ES) for 24 h during osteoclast culture on plastic and imaged after DAPI nuclear staining to assess levels of multinucleation. SFX-01 reduces cell size and the number of nuclei comprising a single osteoclast.

The data above support the predominant effect of SFX-01 on osteoclast differentiation, without clear evidence of cell toxicity, which commences at the point when mononuclear osteoclast precursors are initially exposed to M-CSF and RANKL in the first 24 h after plating. To explore how early and persistent these effects of SFX-01 are on osteoclast differentiation, transient SFX01 exposure was only administered in the very earliest stage (ES; D1–D2) of M-CSF and RANKL exposure during the initial 24 h after plating onto dentine. The results show that the initial 24 h of SFX-01 exposure was sufficient to inhibit osteoclastogenesis to a similar extent to that seen with SFX-01 throughout the culture (DC; D1–D7) or later differentiation stages (DS; D2–D5). There were no significant differences found between DS, DC or ES (*p* > 0.05), suggesting that this short and early exposure was sufficient for lasting inhibition (Figure 3A).

As early osteoclast differentiation accommodates vital osteoclast precursor multinucleation, the effects of early exposure to SFX-01 were additionally explored through DAPI staining to assess the levels of multinucleation. These studies indicated that the addition of SFX-01 at the ES generated cells with smaller overall size containing fewer nuclei compared to the controls (treated with M-CSF and RANKL; Figure 3B).

**Figure 4:**
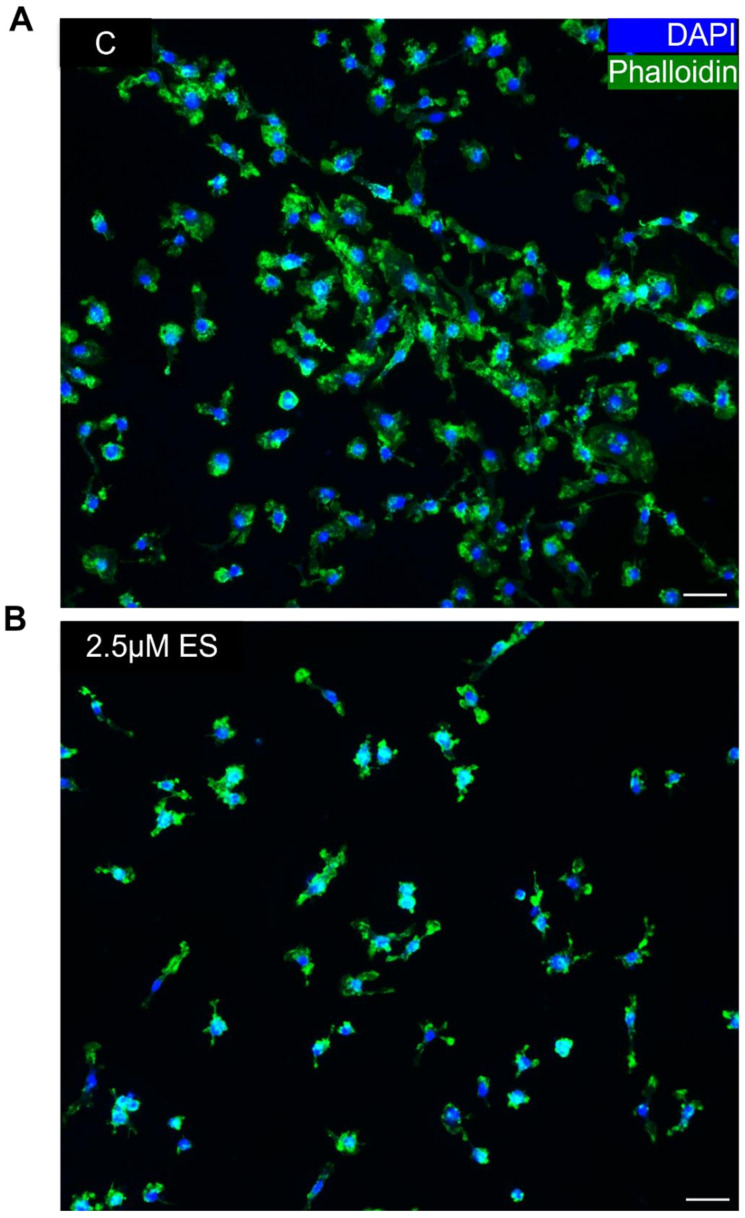
Immunofluorescent labelling of mature OCs treated with SFX-01 in the ES reveals modified polymerised actin organisation. Osteoclasts cultured for 6 days and then immunocytochemically labelled with phalloidin (for polymerised actin, green) and DAPI (for DNA, blue) and imaged by confocal microscopy indicating the difference in cytoskeletal architecture in untreated cells (**A**) and cells treated with SFX-01 (**B**).

**Figure 5:**
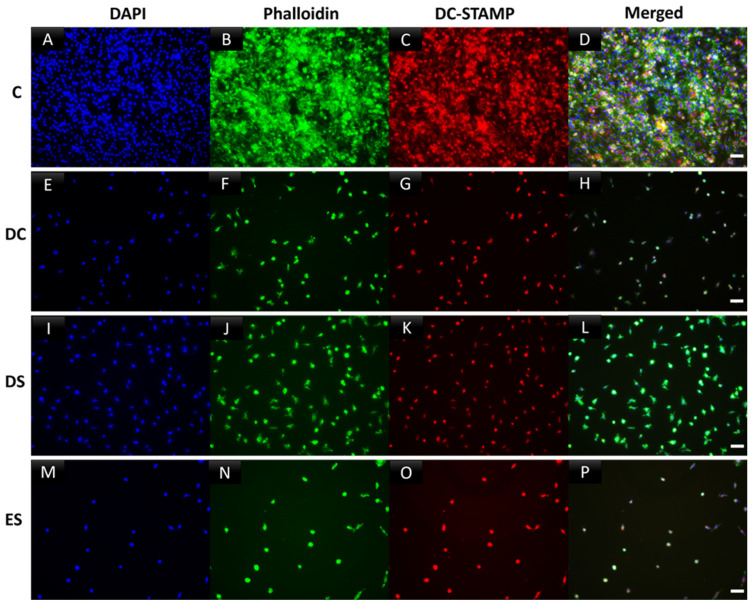
Incubation with SFX-01 indicates inhibition of cell–cell fusion/multinucleation in osteoclasts grown on plastic. Osteoclasts cultured for 6 days and then immunocytochemically labelled for polymerized actin (phalloidin, green), DC-STAMP (red) and nuclear DNA (DAPI, blue). Control cultures showed positive labelling for cytoskeletal actin, cell spreading, cell fusion and multinucleation (**A**–**D**). These features were inhibited by the addition of SFX-01 at all timepoints, but particularly prominently at ES and DC (**E**–**H**,**M**–**P**). DS treatment likewise inhibited cell fusion (**I**–**L**) but less markedly.

This multinucleation, cell–cell fusion also involves morphological changes in cell architecture. To determine whether SFX-01 modifies the morphological changes involved in multinucleation, cells were cultured on plastic and labelled for polymerised cytoskeletal actin using phalloidin and for nuclear DNA using DAPI, with or without early prior SFX-01 exposure during the early stage. Visualisation of the control cultures revealed overt cytoskeletal spreading and the presence of cellular phalloidin-positive polymerized actin in osteoclasts (Figure 4A) and the complete absence of actin polymerisation in SFX-01 treated cultures (Figure 4B). The effects of SFX-01 on cell–cell fusion in the ES were further assessed by triple-labelling for polymerised actin, nuclear DNA and for DC-STAMP using selective antibody labelling. A visualisation of the labelling indicated a diffuse extra-nuclear distribution of DC-STAMP that co-localised with polymerised actin in control cells where multinucleation was supported by clusters of DAPI-stained nuclei (Figure 5A–D). In contrast, cells exposed to SFX-01 during the DC, ES or DS only exhibited nuclear labelling for DCSTAMP, showed no co-localisation with polymerised actin (that was absent in these cells) and failed multinucleation (Figure 5E–P). The similarity in the outcomes of SFX-01 treatment in cells exposed during the DC, ES and DS confirms that the inhibitory actions of SFX-01 start during the early phase (Figure 5M–P).

### 3.3. NRF2 and NF-KB Activators Inhibit Osteoclastogenesis

**Figure 6:**
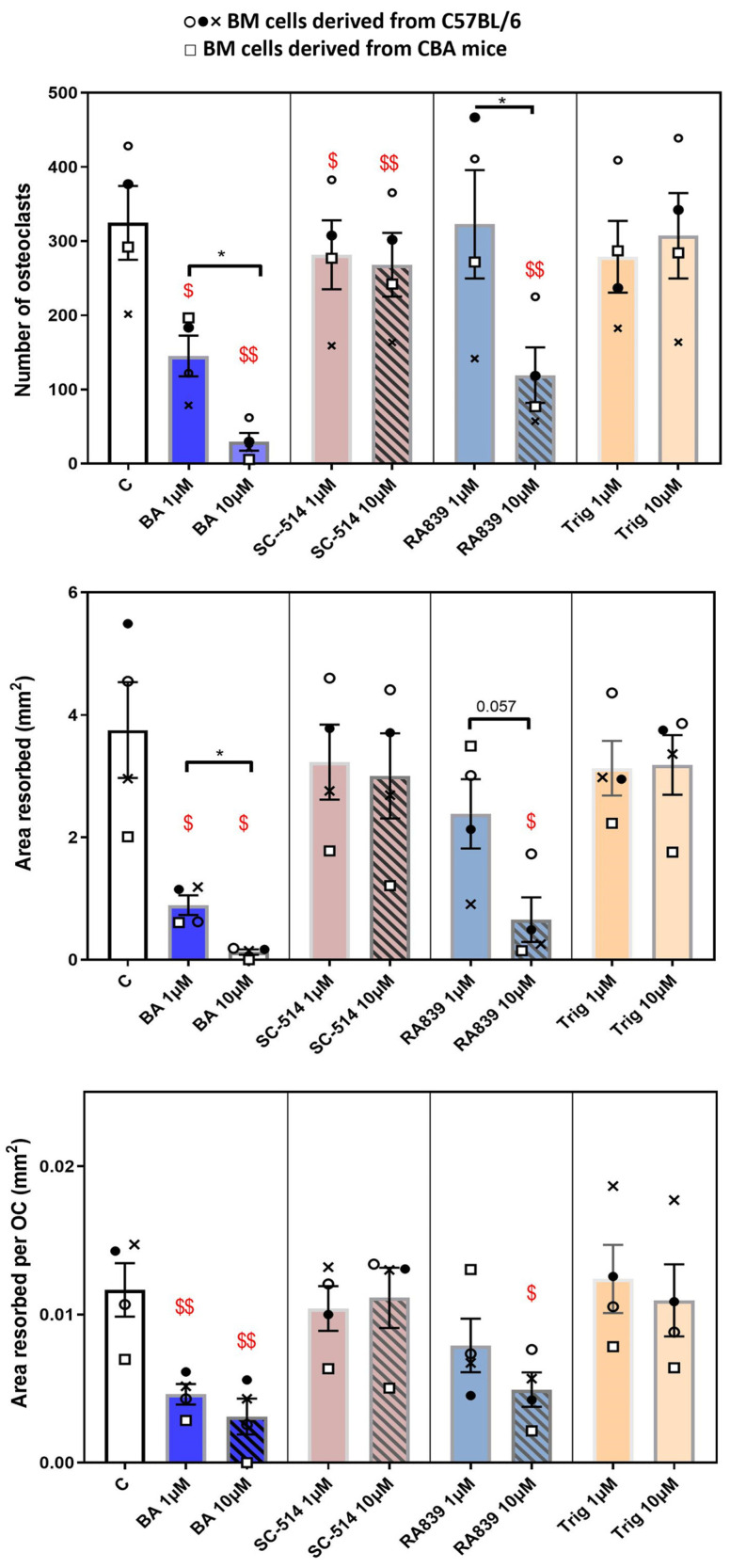
Regulation of osteoclast behaviour by NRF2 and NF-KB modulators. RA839 (NRF2 activator) and BA (NF-KB activator) exert the most marked inhibitory effects on osteoclast formation/resorption when added for the duration of culture (7 days). TRAP+ osteoclast numbers, area resorbed, area resorbed/OC (mean ± SEM). $ and $$ denote *p* ≤ 0.05 and *p* ≤ 0.01 difference from control. * denotes *p* ≤ 0.05 differences between 1 μM and 10 μΜ for each compound. Combined data from *n* = 5 experiments, 6–8 replicates/condition. Open/closed circles and crosses denote BM cells from C57/Bl6 and squares CBA, respectively.

This section seeks to identify whether pharmacological activators/inhibitors of the NRF2 and NF-KB pathways independently affect osteoclastogenesis. The effects of the alkaloid trigonelline (Trig), a recognized inhibitor of NRF2, and RA839, an activator of NRF2, on osteoclastogenesis and resorption have not previously been investigated. These studies were performed in osteoclasts from C57BL/6 and CBA mice, which we have found display a greater sensitivity to exogenous anti-osteoclastogenic factors. All activators/inhibitors were each added at 1 and 10 μΜ. Trends were identical in cells from both mouse strains and these data were combined for statistical comparison. These studies showed that the NRF2 inhibitor, Trig, did not affect osteoclast number, total area resorbed or area resorbed/osteoclast (Figure 6). In contrast, 10 μM RA839 (NRF2 activator) inhibited osteoclast number, total area resorbed and area resorbed/osteoclast by more than 50%.

Similar studies performed showed the NF-KB activator betulinic acid (BA) limited osteoclastogenesis and resorption in a dose-dependent manner (Figure 6). This suggests that NF-KB activation restricts osteoclast formation and resorption and, thus, it may be predicted that the NF-KB inhibitor, SC-514 may have opposing effects. The differentiation of osteoclasts in medium supplemented with SC-514 did not, however, conform to this simple expectation; instead, SC-514 caused a mild, yet significant, inhibition of osteoclast number, but had no effects on total or per osteoclast resorption.

### 3.4. Co-Administration of NRF2 Activator/Inhibitor Does Not Further Augment the Effects of SFX-01

**Figure 7:**
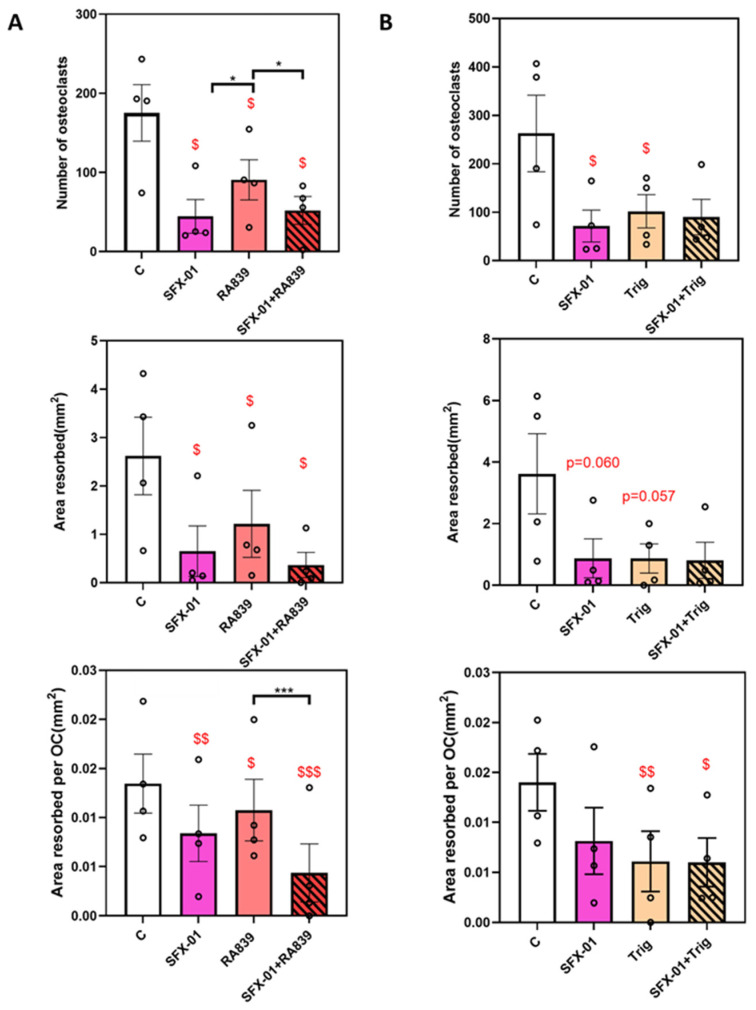
Regulation of osteoclast behaviour by NRF2 and SFX-01. (**A**) RA839 (NRF2 activator) inhibits osteoclast formation/resorption in the early stage (ES; D1–D2) but there is no additional inhibition exerted when SFX-01 is co-administered; indicating that inhibition by SFX-01 is stronger than RA839. (**B**) Trig (an NRF2 inhibitor) also inhibits osteoclast formation/resorption at early stage (ES; D1–D2); no additional inhibition with SFX-01. TRAP+ osteoclast numbers (**top**), area resorbed (**centre**) and area resorbed/OC (**bottom**, mean ± SE) from STR/Ort mice. $, $$ and $$$ denote *p* ≤ 0.05, *p* ≤ 0.01 and *p* ≤ 0.001 from the control; * *p* ≤ 0.05, *** *p* < 0.001 denote statistical difference between the different groups. Combined data from *n* = 4 experiments, with 6–8 replicates/condition.

Treatment during only the early phase (D1–D2, ES) in cells derived from STR/Ort mice showed that RA839 (NRF2 activator) reduces osteoclast number. However, this effect was less potent than SFX-01 (Figure 7A), and there is no additional inhibition by RA839 in the presence of SFX-01. In contrast with previous studies (Figure 2A, Figure 3A and Figure 6), early-stage SFX-01 exposure also significantly reduced the area resorbed/osteoclast. Nonetheless, there was a lack of any additional inhibition caused by RA839 on either the total area resorbed or the area resorbed/osteoclast in the presence of SFX-01.

If the effects of SFX-01 on osteoclastogenesis are solely mediated by NRF2 activation it would be expected that the NRF2 inhibitor Trig would block the actions of SFX-01. However, Trig alone reduced osteoclast number and area resorbed/osteoclast (Figure 7B). When Trig was combined with SFX-01, there was a clear a lack of any additional inhibition exerted by the inhibitor on any of the measured parameters. This suggests that the mechanism of SFX-01’s action upon osteoclastogenesis and resorption may involve modulation of the NRF2 pathway.

**Figure 8:**
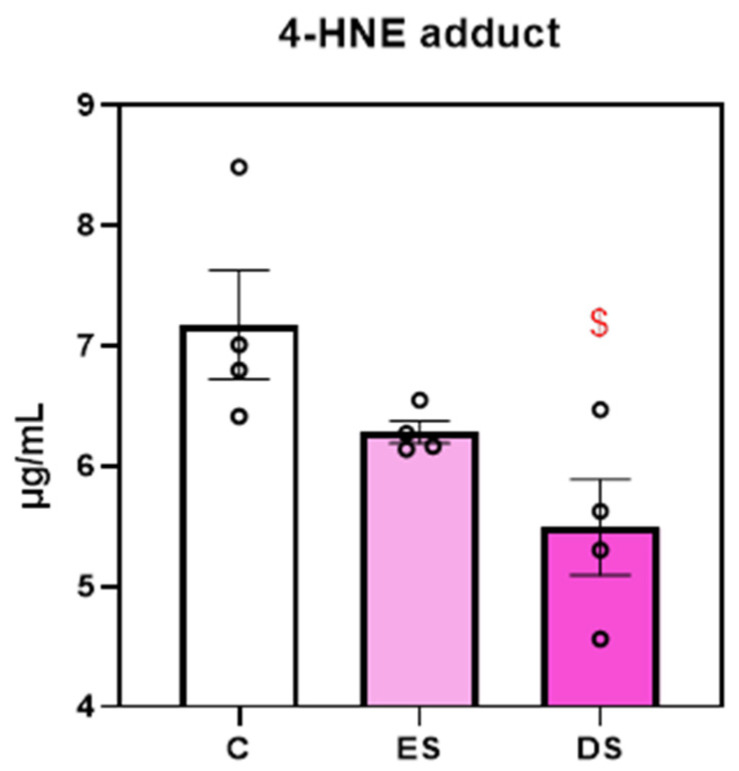
Assay for the 4-HNE adduct indicates that cultures treated with SFX-01 (DS; D2–D5) expressed lower oxidative stress levels than the untreated cells. $, denote *p* ≤ 0.05 from the control.

To explore whether SFX-01 modulates oxidative stress, the 4-HNE adduct was measured using ELISA in medium collected from osteoclast cultures treated at different stages with SFX-01. These data show that the collection, on day 7, of the medium conditioned by osteoclast cultures treated with SFX-01 during differentiation stages (DS, D2–D5) expressed lower 4-HNE levels, compared to control-vehicle-treated cells, while those treated only during the ES (D1–D2) show less marked changes that did not reach levels of significance (Figure 8).

### 3.5. Co-Administration of an NF-KB Activator but Not Inhibitor Augments SFX-01 Effects of SFX-01

**Figure 9:**
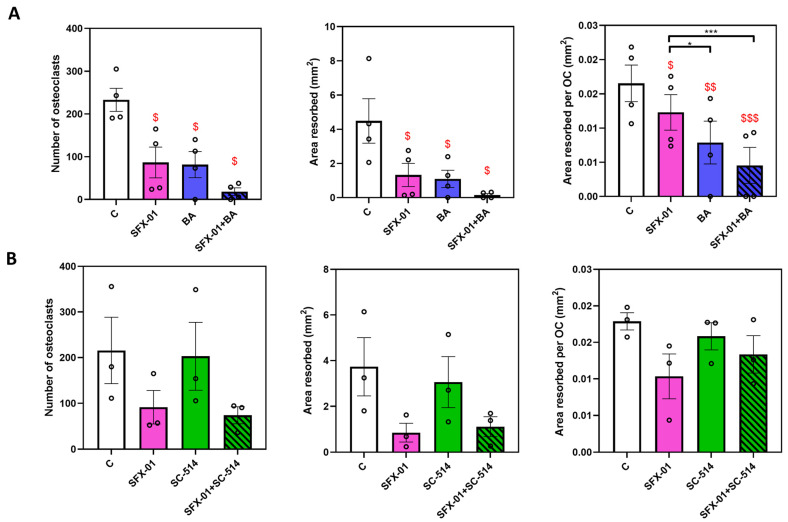
Regulation of osteoclast behaviour by NF-KB and SFX-01. (**A**) BA (NF-KB activator) inhibits osteoclast formation/resorption at early stage (ES; D1–D2); when supplemented with SFX-01 there was no additional inhibition greater than either with BA or SFX-01 alone. (**B**) SC-514 (an NF-KB inhibitor) did not modify osteoclast formation/resorption at early stage (ES; D1–D2) alone with SFX-01. TRAP+ osteoclast numbers (**left**), area resorbed (**centre**) and area resorbed/OC (**right**, mean± SE) from STR/Ort mice. $, $$ and $$$ denote *p* ≤ 0.05, *p* ≤ 0.01 and *p* ≤ 0.001 from the control. * *p* ≤ 0.05, *** *p* < 0.001 denote statistical difference between different groups.

Treatment with 1 μΜ BA, the NF-KB activator, during only early (D1–D2, ES) stages restricted osteoclastogenesis and resorption (Figure 9A), with osteoclast number, total resorbed area and area resorbed/osteoclast being lowered by more than 50% (vs. controls). Intriguingly, combined BA/SFX-01 treatment generated significantly lower area resorbed/osteoclast, but only modestly lower levels of total osteoclast numbers and area resorbed (non-significant) than with SFX01 alone. SC-514 (10 μM) did not show any effect on osteoclast formation or resorption (Figure 9). Likewise, combined SC-514 and SFX-01 treatment failed to modify levels of osteoclast formation and resorption seen in cultures treated with SFX-01 alone, indicating that SC-514 did not have any effect either in the presence or absence of SFX-01.

## 4. Discussion

These data show that SFX-01 preferentially, rapidly and irreversibly targets the early osteoclastogenic stages by inhibiting the cell–cell fusion and cell spreading required for multinucleation. Based on the use of pharmacological agents, it appears that SFX-01 appears to achieve these effects, akin to SFN, via the NRF2 pathway. These are the first data exploring the mechanism by which SFX-01 influences osteoclastogenesis.

Herein, SFX-01 can target all stages of osteoclastogenesis but, in contrast to known bisphosphonate anti-resorptive compounds which target osteoclast resorptive activity, exerts the most profound actions on early osteoclast precursor differentiation to exert lasting restriction. This aligns with previous findings for SFN, showing that it similarly targets osteoclastogenesis and specifically decreases the osteoclast formation rate, with fewer TRAP+ cells [27,28,35] and a lower expression of osteoclast differentiation-associated genes such as *NFATc1*, *NF-KB*, *c-Fos*, *DC-STAMP* [28]. These conclusions regarding SFN are based on the seeding of cells (RAW 264.7 or BMCs) onto plastic plates, and thereby, any possibility that SFN might directly influence resorption was negated. Only one study by Kim et al., (2005) describes assays using dentine to explore resorption, but no data for these assays are shown. To determine which osteoclastogenic stage was targeted by SFN, lymphocyte-free precursors were seeded on plastic plates and SFN was added at different timepoints to reveal that a minimum 3 days of exposure was required for inhibition [27]. This aligns somewhat with the data herein, in which it has been shown for the first time that 7 days, 3 days or as little as 24 h (1 day) of SFX-01 treatment was sufficient for effective inhibition.

Mononuclear osteoclasts promote cell–cell fusion by increasing the expression levels *of* the fusogens *DC-STAMP* or *OC-STAMP*, which ultimately leads to osteoclast formation and bone resorption [36]. Additionally, it has been shown that Atp6v0d2-deficient mice exhibit impaired multinucleation emphasising its importance in cell fusion [37]. SFN decreases osteoclast cell fusion genes such as *DC-STAMP*, *OC-STAMP*, and *Atp6v0d2* in sRANKL-induced multinucleated osteoclasts [28,35] and osteoclasts appear to be smaller in size with fewer nuclei. Additionally, the immunohistochemical data presented herein offer support for the notion that SFX-01 affects fusion/multinucleation, with some evidence indicating a decrease in the cellular levels of DC-STAMP and reduced labelling for polymerised actin.

There are many studies indicating that NRF2 plays an important role in bone cell regulation and homeostasis. Global NRF2 deletion increases RANKL expression and thereby increases number of osteoclasts and resorption [29,38,39,40]. Within this context, it is established that SFN activates NRF2, which, via binding to the antioxidant response element in cognate genes, serves to upregulate protective enzymes with known anti-inflammatory roles [17]. There are several methods available for measuring ROS. Herein, we quantified oxidative stress by assessment of the lipid peroxidation marker, 4-HNE. The results indicated that exposure of cells to SFX-01 also decreased levels of oxidative stress in addition to lowering levels of osteoclastogenesis. This indicates an SFX-01-induced modulation of oxidation status and suggests that this occurs through NRF2 activation.

As a prelude to examining whether SFX-01 also achieves its effects on osteoclasts via the modulation of NFR2, our studies were initially focused on whether pharmacological NRF2 modulators, namely Trig and RA839, exerted direct effects on osteoclast formation and function. The alkaloid, Trig, has been used in many studies as an inhibitor of NRF2 in other cell types [41,42,43]. On the other hand, RA839 has only recently been characterised as a non-covalent KEAP1 binding partner and selective activator of NRF2 [44]; neither have been used in assays of osteoclastogenesis previously. Initially we used each at both 1 and 10μM and found that Trig (an NRF2 inhibitor) did not modify osteoclastogenesis or resorption in BM precursors, but intriguingly, that RA839 (NRF2 activator) significantly inhibited osteoclast formation and osteoclast resorption/cell when administered at 10 μM. These data imply that direct NRF2 activation with RA839 abrogates all osteoclast maturation and resorptive phases extending to the activity of individual osteoclasts, but that inhibition of NRF2 (Trig) is insufficient to stimulate osteoclastogenesis or resorption, under these assay conditions. These data align to some extent with the findings that SFX-01 achieves its inhibition via the activation of this NRF2 pathway.

To further explore interaction between SFX-01 and direct NRF2 pathway modulation, the effects of RA839 (10 μΜ) were examined in the presence and absence of known inhibitory concentrations of SFX-01 (2.5 μΜ). As previous data had defined early stage SFX-01 supplementation sufficient for inhibition, all compounds were added only for the initial 24 h (ES). This confirmed that RA839 administration, in the absence of SFX-01, reduced osteoclast number and area resorbed/osteoclast in BM precursors. The combined addition of SFX-01/RA839 to BM cultures revealed similar inhibition levels in osteoclastogenesis and resorption achieved through the administration of SFX-01 alone, implying that the pharmacological NRF2 activator was rendered ineffective by the presence of SFX-01. These results suggest that SFX-01 likely exerts its action via activation of NRF2 to inhibit osteoclastogenesis and total resorptive function.

The results for Trig (an NRF2 inhibitor) are more difficult to interpret. Whilst without any measurable effect even at 10 μΜ on cells derived from C57BL/6 and CBA mice, Trig instead significantly inhibited both osteoclast formation and resorption/OC in BM cells derived from STR/Ort mice. These differences may be explained by different mouse strain-related sensitivities based upon bone mass [26]. Nonetheless, there were no additional adjunct activities exerted by Trig over those exerted by SFX-01 alone in this study, suggesting that Trig was ineffective when combined with SFX-01. The possibility that the NRF2-inhibitory functions of Trig may remain cryptic, unless combined with an NRF2 activator, in this case SFX-01, would, however, require much more detailed pharmacological profiling. Likewise, it remains possible that Trig may not be very effective as an inhibitor of NRF2 under the conditions of these assays.

Treatment of BM cells with BA (NF-KB activator) revealed a concentration-dependent inhibition of osteoclast formation and resorption when added for the duration of the culture (DC). This aligns with prior studies that have found that BA suppresses osteoclast formation and acts as an inhibitor of bone resorption [45,46]. Nonetheless, this is somewhat in conflict with the notion that NF-KB activation is required for OC formation; further studies into the actions of BA are required to resolve these apparent conflicts. Similar studies performed using SC-514, an NF-KB inhibitor, showed that it also restricted osteoclastogenesis without modifying resorption, even though one might predict, based upon data from BA, that this NF-KB inhibitor would have opposing effects [47].

To explore the interaction between the effects of SFX-01 and direct NF-KB pathway modulation, the effects of BA (10 μΜ) were examined with and without SFX-01 (2.5 μΜ) supplementation only for the initial 24 h (ES). The treatment of mouse cells consistently indicated that BA more efficiently targets area resorbed/osteoclast than it does osteoclastogenesis compared with SFX-01. Treatment with SC-514 failed to exert statistical differences in osteoclast behaviour; albeit using a relatively small sample size.

It is important to emphasise that these studies explore the actions of stable SFX-01, with a strong focus upon the NRF2 and NF-KB pathways that are most strongly implicated in mediating the effects of its unstable counterpart SFN. It is important to stress that the NRF2 and NF-KB pathways are not the only potential targets for SFN in osteoclasts. Recent studies indicate that SFN may also inhibit osteoclastogenesis by suppressing the autophagic pathway, with fewer autophagosomes in RAW 264.7 cells and attenuation in autophagy markers [34]. The simultaneous addition of rapamycin, an mTOR inhibitor that activates autophagy, was capable of reversing the inhibition of osteoclastogenesis induced by SFN [34]. The possibility that SFX-01 may also achieve its effects via these mechanisms has not, however, been explored. It is also feasible that the chemical stabilisation of SFN, SFX01, may allow alternative mechanisms to be targeted. These would perhaps be best identified by some non-targeted screening; the studies described here serve to both verify a cellular, osteoclast precursor target for SFX-01’s actions and also define timeframes over which exposure to SFX-01 could be explored. In conclusion, this study shows that SFX-01 preferentially and more effectively targets the early stages of osteoclastogenesis rather than resorption and that it does so relatively rapidly and irreversibly. We also identified prominent roles for NRF2 activation over NF-KB pathway involvement in SFX-01-related inhibition of osteoclastogenesis.

## 5. Conclusions

This study has shown that SFX-01 preferentially and more effectively targets the early stages of osteoclastogenesis rather than resorption and that it does so relatively rapidly and irreversibly. The data also imply support roles for NRF2 activation much more strongly than they do roles for NF-KB involvement in SFX-01-related inhibition of osteoclastogenesis.

## Data Availability

Data are available on request from the authors.
